# Temporal Variability of Gallium in Natural Plants

**DOI:** 10.3390/toxics11080675

**Published:** 2023-08-07

**Authors:** Irina Shtangeeva

**Affiliations:** Institute of Earth Sciences, St. Petersburg University, St. Petersburg 199034, Russia; shtangeeva@gmail.com; Tel.: +7-8126876122

**Keywords:** gallium, grasses, rhizosphere soil, phytoextraction, short-term variability

## Abstract

The aim of the research was to study the distribution of gallium (Ga) in rhizosphere soil and in plants growing under natural conditions in uncontaminated sites, with an emphasis on temporal fluctuations of Ga concentration in plants. For this purpose, two field experiments were conducted in St. Petersburg, Russia, in 2019 and 2020, at two sites. Three widespread grasses (couch grass, plantain, and dandelion) were chosen for the experiments. ICP–MS analytical technique was applied for the determination of Ga. All plants were capable of accumulating Ga, but the uptake of Ga was different in different plant species, although the plants grew under the same conditions. It can be assumed that one of the main reasons for such differences was the belonging of the plants to different botanical classes, where biochemical processes can proceed differently. The concentration of Ga in plants and rhizosphere soil varied in the daytime. The daily fluctuations of Ga in different plant species were often completely different and did not resemble the temporal fluctuations of Ga in rhizosphere soil. These short-term variations were due to natural reasons and should be considered when collecting plant and soil samples.

## 1. Introduction

Gallium (Ga) is a member of the 13th group of the Mendeleev Table. Its closest “neighbors” in the group are aluminum and indium. Gallium is a technology-critical element. It has been widely used in high-tech industrial activities. The two main applications for Ga are integrated circuits and optoelectronic devices [1]. Gallium has a similar ionic radius with elements such as magnesium (Mg), aluminum (Al), manganese (Mn), and iron (Fe) [2]. This may point to a similar behavior of these elements in environmental processes. In particular, it can be assumed that Ga can be sorbed by Fe(III) and Mn(III) oxides and organic matter.

Until now, little was known about the biogeochemistry of Ga. Unfortunately, most of the current scientific reports are usually limited to well-known macronutrients and so-called heavy metals. Gallium is often not included in this short list of elements. A recent experiment showed that Ga might be taken up by plants via Al pathways [3]. However, this experiment was performed in hydroponics, and it is known that the patterns of absorption of elements by plants grown in soil and in liquid media can be very different [4]. Compared to many other trace elements, the mobility of Ga in the soil-plant system has been reported to be low [5]. This means that Ga can accumulate in the soil and be slowly transferred to plants.

Gallium accumulates mainly in roots [6,7]. Since Ga can behave similarly to Al, it has been suggested that it can be stored in the epidermis and outer cortex of roots [5,8]. Thus, Ga retention in root cells probably contributes to the low concentration of trace elements in plant leaves [9]. It can be assumed that only a limited amount of Ga can be transferred to the upper parts of plants.

The analysis of Ga in environmental samples, especially in plants, is a difficult task, since this trace element is usually present in the samples at low concentrations. In our research, Ga was determined using ICP–MS. Due to the high sensitivity of this analytical technique, it is preferred for the analysis of Ga in soil and plant material [10].

To date, no detailed work has been carried out to identify the patterns of temporal changes in Ga in widely distributed natural plants. The aim of the experimental study was to examine the distribution of Ga in rhizosphere soil and plants growing under natural conditions in uncontaminated places. When studying the uptake of Ga by weeds (couch grass, plantain, and dandelion), main attention was paid to the short-term (within hours) fluctuations of Ga in different plant parts and rhizosphere soil. Although these weeds are distributed in different regions, we could not find information on Ga in the plant species.

## 2. Materials and Methods

### 2.1. Experimental Design

Two field experiments were performed in the south of St. Petersburg, Russia (59°53′ N, 30°38′ E). The distance between these two sites was ~500 m. Plants and rhizosphere soil were collected in May 2019 (experiment 1) and May 2020 (experiment 2). Climatic conditions on both dates of sampling were similar: the average temperature was 15 °C (experiment 1) and 16 °C (experiment 2), and there was no precipitation. The age of plants was ~15 days. This time of sampling was chosen because all physiological and biochemical processes in young plants are active. This allows for a more detailed study of the transfer of elements from soil to plants. The soil in the sites was classified as urban podzol with a sandy loam texture (sand 67%, silt 20%, clay 13%) at site 1 and loamy sand texture (sand 74%, silt 24%, clay 2%) at site 2. The size of each site was ~2 × 2 m. Couch grass (*Elytrigia repens* L.) and plantain (*Plantago major* L.) were dominant plants at site 1. Couch grass and dandelion (*Taraxacum officinale* L.) prevailed at site 2. Samples of plants and soil were taken several times during the day: experiment 1, every 4 hours, from 6:00 to 22:00; experiment 2, every 5 h, from 9:00 to 19:00. The soil adhering to the plant roots was taken by brushing it off with a toothbrush. At each collection of samples from both sites, three-to-six plants of each species were collected. The amount of soil taken from the plant roots was ~200 g. 

### 2.2. Analysis of Plant and Soil Material

Immediately after collection, the plants were thoroughly washed with tap water to remove dust and small soil particles from the surface of the roots and leaves, and dried at room temperature to a constant weight. Each sample was weighed into a Teflon microwave digestion vessel. Then 8 mL of concentrated HNO_3_ was added, the vessels were closed, and the samples were heated in the Millestone microwave oven (program: heating for 15 min to 130 °C and holding at 130 °C for 30 min). After the program was completed, the vessels were cooled down to room temperature. Then, the samples were diluted to 20 mL with Millipore deionized water. An inductively coupled plasma mass spectrometer (ICP–MS, Agilent 8900 ICP-QQQ, Santa Clara, CA 95051, USA) equipped with a micro-mist nebulizer and a collision/reaction He cell was used to determine Ga. A description of the procedure for ICP–MS analysis is given in our previous publication [11]. The quality of the analytical procedure was assessed by determining Ga in the certified reference material (CRM) BCR-060-Aquatic plant (*Lagarosiphon major*) provided by the IRMM (Geel, Belgium) and in the CRM Tomato leaves 1573a (National Institute of Standards and Technology, Gaithersburg, MD, USA). The differences between measured and certified/informative values did not exceed 10%. At least three replicates of each sample were used for the analysis.

### 2.3. Data Analysis

For statistical analysis, Statistica for Windows, version 8.0 Software packages (StatSoft, Tulsa, OK, USA) were applied. The mean concentrations of Ga were calculated and an analysis of variances to estimate statistically significant differences between the groups of samples was conducted. The level of significance was set at *p* < 0.05. The normality of variances of the data was checked using the Shapiro–Wilk test. Pearson correlation analysis was used to study the relationship between Ga concentrations in plants and rhizosphere soil, as well as between concentrations of Ga in different parts of plants.

## 3. Results and Discussion

### 3.1. The Experiment Performed at Site 1

Table 1 shows the mean concentrations of Ga in couch grass and plantain and in the soil taken from the roots of plants. The Ga concentration in rhizosphere soil of couch grass and plantain was similar. As reported, the range of the Ga concentrations in soils is quite wide, from 3 to 300 mg kg^−1^ [5,12,13,14]. Compared to these concentrations, the level of Ga in the experimental soil was quite low. Thus, it can be assumed that the soil at the site was not contaminated with Ga. 

The recorded concentrations of Ga in plants growing in uncontaminated soils range from 0.001 to 0.30 mg kg^−1^ [2,5,9,14,15,16]. Most of the published data relate to cultivated plants such as rice and wheat. To date, little is known about the concentration of Ga in various weeds. Ha et al. [17] studied the distribution of metals (including Ga) in 10 grasses. The range of the Ga concentrations in the roots of plants was from 0.34 to 8.70 mg kg^−1^, and in leaves from 0.21 to 6.75 mg kg^−1^. The foliar concentration of Ga in perennial ryegrass (*Lolium perenne* L.) could reach 11.6 mg kg^−1^ [5]. The concentration of Ga in the roots and leaves of couch grass and plantain is within the published data. However, it should be remembered that comparing the data on plants growing under different conditions can be difficult because there are various factors that can affect the uptake of elements by different plant species. It is hardly possible to take into account all these factors for a correct comparison.

The concentration of Ga was much higher in rhizosphere soil than in the roots of both plants, and the lowest Ga concentration was observed in leaves. The correlation between Ga concentration in rhizosphere soil and the roots of plantain was statistically significant (r = 0.60), while in couch grass, this correlation was insignificant. On the other hand, in couch grass, the correlation between Ga concentrations in roots and leaves was statistically significant (r = 0.58), and there was no correlation between Ga concentrations in the roots and leaves of plantain. 

Although couch grass and plantain grew in the same place, were harvested at the same time, and the concentrations of Ga in the rhizosphere soil of the plants were similar, concentrations of the trace element in the plants differed significantly (*p* < 0.05). The mean concentration of Ga in the roots of couch grass was much higher compared to the Ga concentration in the roots of plantain. In the leaves of couch grass, the Ga concentration was lower than in the plantain leaves. These significant differences between Ga concentrations in the two plants may be due to the fact that couch grass and plantain are in different botanical classes: couch grass is a monocot and plantain is a dicot. It is known that there are significant differences in physiological and biochemical characteristics of monocots and dicots [18,19].

**Figure 1:**
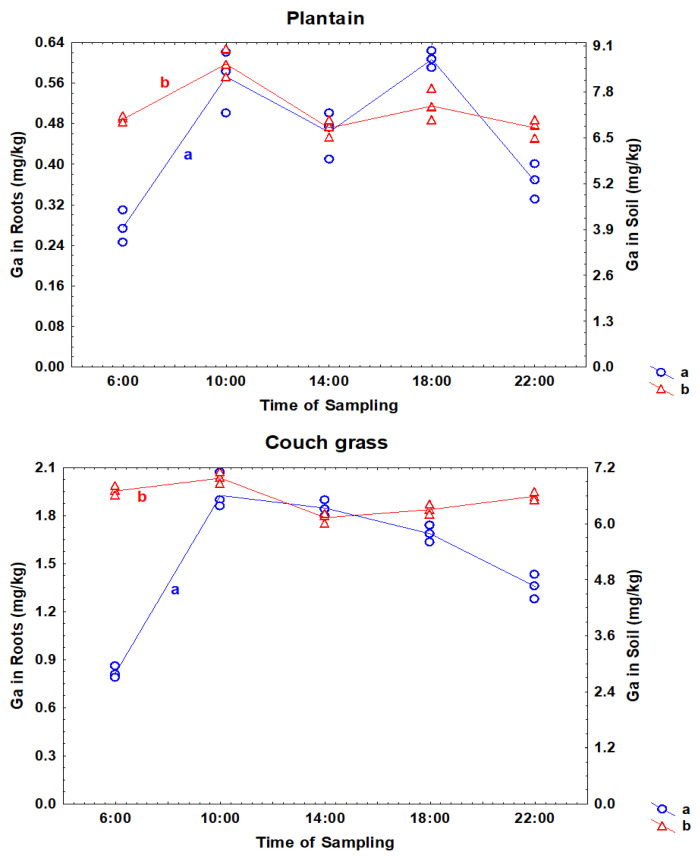
Daily variations of Ga in the roots (a) and rhizosphere soil (b) of plantain and couch grass.

The concentration of Ga in plants, as well as in rhizosphere soil, was not stable during the day. Figure 1 shows the daily fluctuations of Ga in roots and in the soil taken from the roots. During the day, the concentration of Ga in rhizosphere soil could change by 20%, and in roots of the plants by more than two times. These short-term changes in the element concentration in plants and rhizosphere soil may be a consequence of the regular rotation of our planet [20]. It is interesting that in plantain, the daily variations of Ga in rhizosphere soil and roots were similar, while in couch grass, from the middle of the day, the temporal changes in the Ga concentrations in the roots and soil differed. 

**Figure 2:**
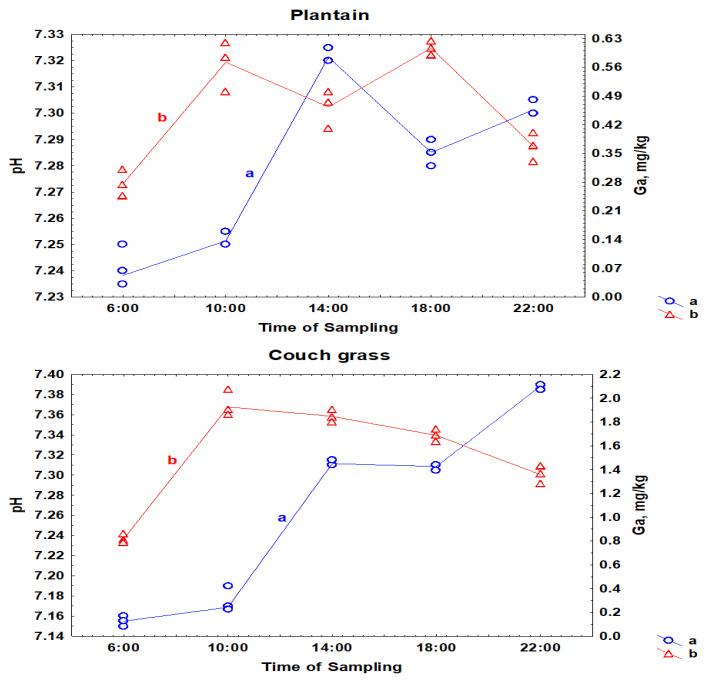
Daily variations in pH of the rhizosphere soil (a) and the concentrations of Ga in the roots of plantain and couch grass (b).

Many researchers have reported that the pH of rhizosphere soil can affect the ability of plants to uptake elements [21,22]. Figure 2 illustrates the relationship between the accumulation of Ga in the roots of plantain and couch grass, and the pH of the soil taken from the roots of plants. Daily changes in Ga concentration in the roots of plantain were opposite to the temporal variations in the pH of the rhizosphere soil of plants. In the first half of the day, the interrelationship between soil pH and Ga in the roots of couch grass was positive, but by the end of the day, it changed to negative.

There is no doubt that soil pH near the roots can affect the solubility, mobility, and bioavailability of elements. However, as can be seen from Figure 2, the relationship between these two parameters—rhizosphere soil pH and Ga uptake by roots—may differ for different plant species, even if the plants grow under the same conditions in the same place. It can be assumed that the role of the soil pH, perhaps, is not the most important in the process of uptake of elements by plants. Most likely, the genetic characteristics of each plant species are of first concern. In particular, the experimental data can serve as additional confirmation of essential differences between biochemical processes occurring in couch grass and plantain.

**Figure 3:**
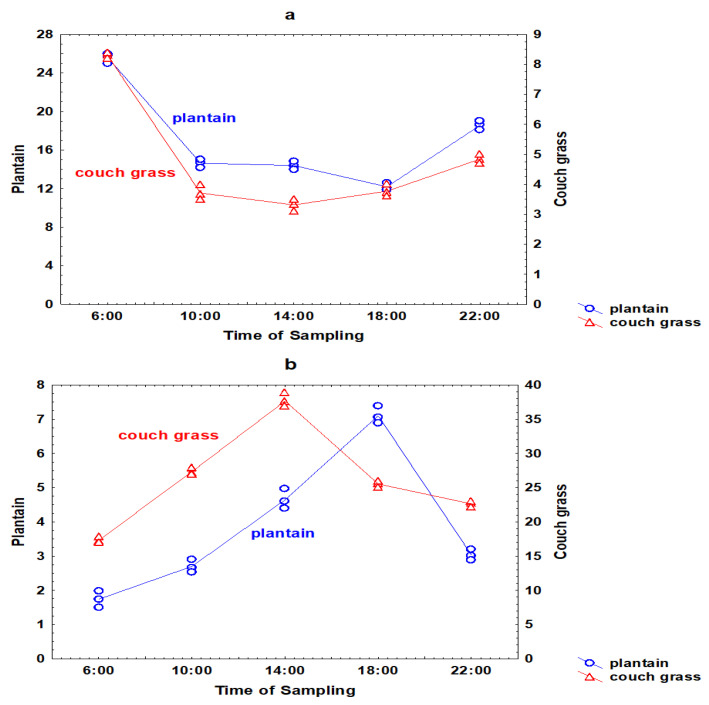
Daily variations of the ratios of the Ga concentration in rhizosphere soil to its concentration in the roots, (**a**) and the ratios of the Ga concentration in the roots to its concentration in the leaves (**b**) in plantain and couch grass.

Ratios of the concentration of an element in the roots to its concentration in the leaves, as well as ratios of the element concentration in the rhizosphere soil to its concentration in the roots, can furnish insights into the patterns of the element transport between these different systems. It would be interesting to trace the redistribution of Ga from rhizosphere soil to the roots and leaves of couch grass and plantain. For this, the ratios of the Ga concentration in the soil taken from the roots of plants to its concentration in roots, as well as the ratios of Ga in roots to its concentration in the leaves of couch grass and plantain, were calculated (Figure 3). The ratios of the Ga concentration in soil-to-roots in plantain were much higher than in couch grass (Figure 3a). The ratios varied during the day and were similar for both plants. The ratios of the Ga concentration in roots-to-leaves were higher in couch grass. The ratios in both plant species demonstrated serious daily fluctuations (Figure 3b). Couch grass had the highest root-to-leaf ratio at 14:00, and plantain at 18:00. 

### 3.2. The Experiment Carried out at Site 2

**Figure 4:**
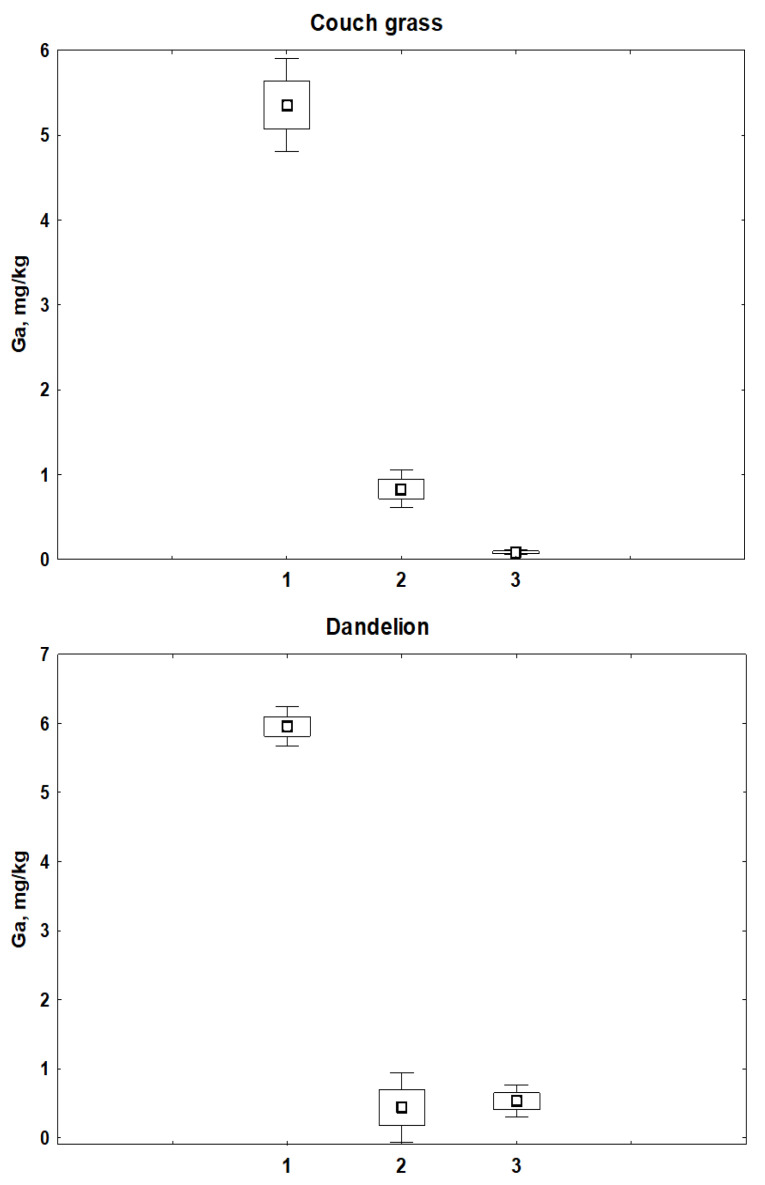
Mean concentrations of Ga in the rhizosphere soil (1), roots (2), and leaves (3) of couch grass and dandelion.

Figure 4 illustrates the distribution of Ga in the roots and leaves of couch grass and dandelion and in the rhizosphere soil of the plants. The concentration of Ga in the soil taken from roots was much higher than in plants. One could also expect a lower concentration of Ga in leaves than in roots, since plants usually accumulate larger amount of many elements in roots [23,24]. In couch grass, the concentration of Ga in leaves was indeed statistically significantly (*p* < 0.05) lower compared to its concentration in roots, but the concentration of Ga in roots and leaves of dandelion was almost the same. Couch grass and dandelion have different anatomical and physiological characteristics. This can lead to differences in plant development, as well as varying ability of the plants to uptake elements from the soil, with the result that each plant species may respond differently to environmental cues. 

Although couch grass and dandelion grew in the same small site under the same conditions and were collected simultaneously, the concentrations of Ga in the leaves of the plants and soil taken from their roots were statistically significantly (*p* < 0.05) higher in dandelion than in couch grass. On the other hand, differences between Ga concentration in the roots of plants were statistically insignificant (0.80 ± 0.15 mg kg^−1^ in couch grass and 0.47 ± 0.26 mg kg^−1^ in dandelion). The correlation between Ga in the rhizosphere soil and roots of dandelion, and between concentrations of Ga in the roots and leaves of the plant was significant (r = 0.59 and r = 0.73, respectively). Alternatively, in couch grass, the relationship between Ga in the soil taken from roots and in the roots of plants, as well as between Ga in the roots and leaves, was not statistically significant.

**Figure 5:**
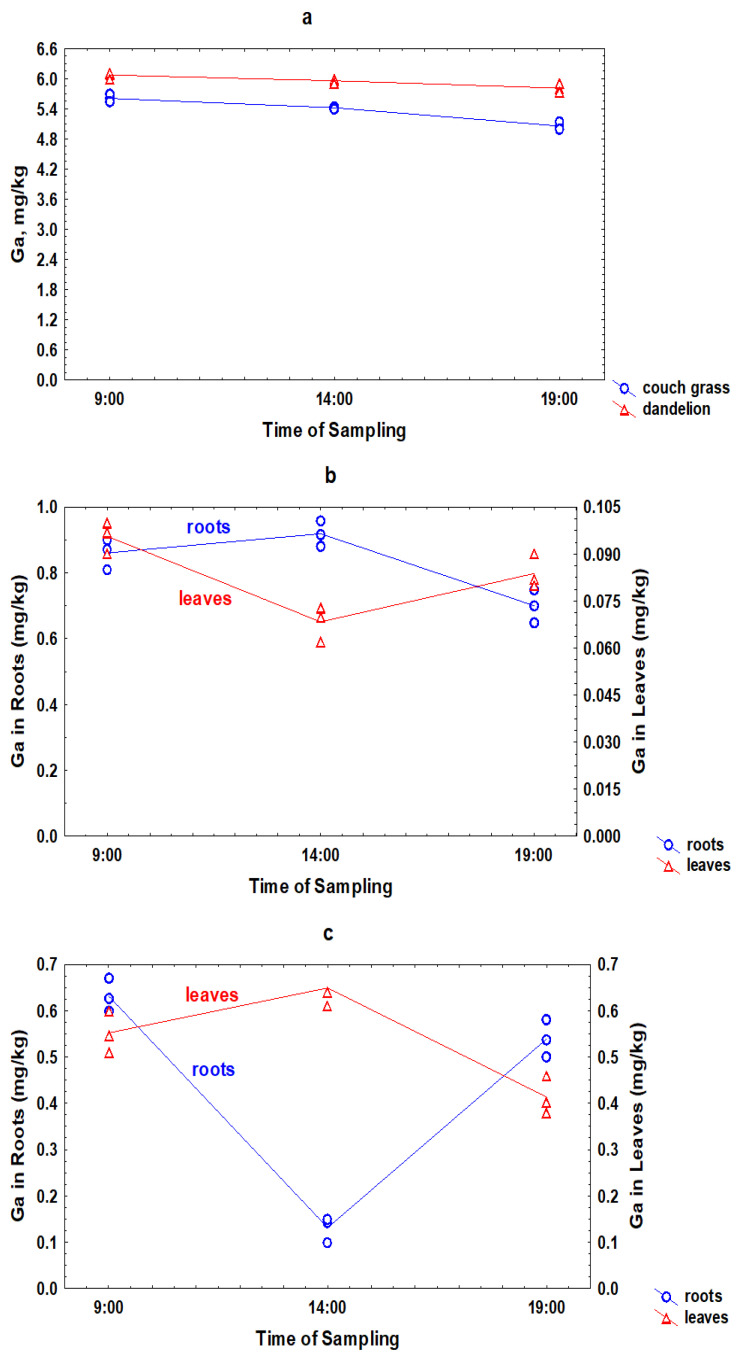
Daily variations of Ga in the rhizosphere soil (**a**), roots (1), and leaves (2) of couch grass (**b**) and dandelion (**c**).

During the day, the concentration of Ga in the rhizosphere soil of couch grass and dandelion decreased (Figure 5). The short-term changes in Ga concentration in plants were significant and not similar to the temporal variations of Ga in rhizosphere soil. It is interesting that the daily fluctuations of Ga in couch grass and dandelion were absolutely different. Although in both plants, an increase in the concentration of Ga in the roots led to a decrease in its concentration in the leaves, the change in the Ga concentration in couch grass was exactly the opposite to that in dandelion. It can be assumed that the main reason for such differences is that the plants belong to different botanical classes: couch grass is a monocot and dandelion is a dicot. As a result, various biochemical processes in the plants can proceed in different ways.

## 4. Conclusions

The field experimental study showed that plants growing in uncontaminated soils are able to uptake fairly high concentrations of Ga. However, the level of Ga in plants has always been significantly lower than in soil. The accumulation of Ga depended on the plant species and could be different in different plants growing under the same conditions within one small site. It has been suggested that the main reason for such differences lies in the belonging of plants to different botanical classes, which leads to serious differences in biochemical processes. The concentration of Ga in different plant species, as well as rhizosphere soil, can vary significantly during the daytime. It was assumed that these fluctuations are quite natural and may be associated with circadian variations.

## Figures and Tables

**Table 1:** Mean concentrations (mg kg^−1^) ± SD of Ga in the roots and leaves of couch grass and plantain and in rhizosphere soil of the plants collected from site 1.

	Couch Grass	Plantain
Soil	6.55 ± 0.33	7.32 ± 0.73
Roots	1.52 ± 0.45 *	0.46 ± 0.14
Leaves	0.058 ± 0.010 *	0.14 ± 0.05

* Differences between concentrations of Ga in couch grass and plantain are statistically significant (*p* < 0.05).

## Data Availability

Not applicable.
